# Results of a Prospective Study of High-Dose or Conventional Anthracycline-Cyclophosphamide Regimen Plus Radiotherapy for Localized Adult Non-Hodgkin's Primary Bone Lymphoma

**DOI:** 10.1155/2014/512508

**Published:** 2014-03-02

**Authors:** A. Schmidt-Tanguy, R. Houot, S. Lissandre, J. F. Abgrall, P. Casassus, P. Rodon, B. Desablens, J. P. Marolleau, R. Garidi, T. Lamy, M.-P. Moles-Moreau, G. Damaj

**Affiliations:** ^1^Hematology Department of the University of Angers, Angers, France; ^2^Hematology Department of the University of Rennes, Rennes, France; ^3^Hematology Department of the University of Tours, Tours, France; ^4^Hematology Department of the University of Brest, Brest, France; ^5^Hematology Department of the University of Bobigny, Bobigny, France; ^6^Hematology Department of the Hospital of Blois, Blois, France; ^7^Hematology Department of the University of Amiens, Amiens, France; ^8^Hematology Department, St. Quentin General Hospital, St. Quentin, France; ^9^University Hospital of Amiens, Department of Clinical Haematology, Avenue Laennec, 80054 Amiens, France

## Abstract

*Background*. Primary bone lymphoma (PBL) is a rare entity that has only been reviewed in one prospective and small retrospective studies, from which it is difficult to establish treatment guidelines. We prospectively evaluated high-dose or conventional anthracycline-cyclophosphamide dose and radiotherapy for PBL. *Patients and Methods*. The GOELAMS prospective multicenter study (1986–1998) enrolled adults with localized high-grade PBL according to age and performance status (PS). Patients <60 years received a high-dose CHOP regimen (VCAP) and those ≥60 years a conventional anthracycline-cyclophosphamide regimen (VCEP-bleomycin); all received intrathecal chemotherapy and local radiotherapy. *Results*. Among the 26 patients included (VCAP: 19; VCEP-bleomycin: 7), 39% had poor PS ≥2. With a median follow-up of 8 years, overall survival, event-free survival, and relapse-free survival were 64%, 62%, and 65%, respectively, with no significant difference between treatment groups. Poor PS was significantly associated with shorter OS and EFS. *Conclusions*. Our results confirm the efficacy of our age-based therapeutic strategy. High-doses anthracycline-cyclophosphamide did not improve the outcome. VCEP-bleomycin is effective and well tolerated for old patients. The intensification must be considered for patients with PS ≥2, a poor prognostic factor.

## 1. Introduction

Primary bone lymphoma (PBL) is a rare clinicopathological entity accounting for about 3% of malignant bone tumors, 1% of non-Hodgkin lymphomas (NHL), and 5% of extranodal NHL [1–4]. In most cases, histology is diffuse large B-cell lymphoma (DLBCL). Whether or not PBL requires specific treatment guidelines has to be determined. Since the 1960s, management of limited stage I-II PBL has usually consisted of radiotherapy to the involved bone and adjacent lymph nodes inducing at least a good local control [2, 5]. Then, adjunction of chemotherapy has systematically been recommended to counter the relatively high rates of relapse occurring outside the original location after radiation alone. For advanced cases, corresponding to disease with multiple bones localizations, treatment can only be based on this combined-modality with generally very good prognosis [1, 6, 7].

To date, PBL has primarily been reviewed in several small retrospective studies and only one prospective study [1, 4, 8–10]. Our prospective study aimed to evaluate overall survival (OS), event-free survival (EFS), and relapse-free survival (RFS) after high-dose or conventional anthracycline-cyclophosphamide regimen for adults with localized non-Hodgkin's PBL before rituximab era.

## 2. Patients and Methods

### 2.1. Study Design and Patient Eligibility

Patients 17–75 years old with localized high-grade PBL were enrolled in a prospective, multicenter GOELAMS study. These patients accounted for 9.3% of the 305 NHL included in the 02 and 03 GOELAMS trials between March 1986 and May 1998 and gave their written informed consent to participate. The histological diagnosis was confirmed on an excision biopsy, in accordance with the previous working formulation criteria [11].

Staging procedures included performance status (PS), differential white blood-cell counts, biochemical analyses (serum lactate dehydrogenase (LDH) and hepatic and renal function tests), thoracic-abdominal-pelvic computed tomography scan, bone-marrow biopsy, and cerebrospinal fluid cytology. Human immunodeficiency virus-positive patients were excluded. The stage was determined according to the Ann Arbor criteria. Bulky disease was defined as a lesion exceeding >5 cm.

### 2.2. Treatment Protocol

Patients <60 years old (GOELAMS 02 trial) received three cycles of the high-dose CHOP regimen (VCAP), as follows: eldisine i.v. 3 mg/m² on day 1, doxorubicin i.v. 60 mg/m² on day 1, cyclophosphamide i.v. 1500 mg/m² on day 1, and oral prednisone 80 mg/m²/d on days 1–5. Patients ≥60 years old (GOELAMS 03 trial) received three cycles of a conventional anthracycline-cyclophosphamide regimen (VCEP-bleomycin), as follows: eldisine i.v. 3 mg/m² on day 1, farmorubicin i.v. 80 mg/m² on day 1, cyclophosphamide i.v. 750 mg/m² on day 2, oral prednisone 50 mg/m²/d on days 1–7, and bleomycin 10 mg on days 1 and 5.

For both trials, each course was repeated every 21 days and intrathecal chemotherapy (methotrexate 15 mg) was administered with each cycle. Since January 1990, all patients have been given granulocyte-colony-stimulating factor between chemotherapy cycles.

One month after completing chemotherapy, every patient received involved-field radiotherapy (total dose: 40 Gy delivered in 20 fractions, 2 Gy/day) over 4 weeks.

### 2.3. Response Assessment and Follow-Up Evaluation

Treatment response was determined by physical examination and biological and radiological workup. In the GOELAMS prospective multicenter study, complete response (CR) was defined as the complete disappearance of all clinical, biological, and radiological evidence of disease (absence of progressive bone lesions). The follow-up included clinical and radiological evaluation every six months.

Survival analyses included OS, EFS, and RFS. OS was calculated from the time of diagnosis until death from any cause. EFS was calculated from time of diagnosis (i.e., study entry) until disease progression, relapse, second malignancy, and death from any cause. RFS was calculated as survival after achievement of CR until relapse or death.

### 2.4. Statistical Methods

Results are expressed as median (range), mean ± SD, or number (%).

Survival curves were calculated using the actuarial Kaplan-Meier method. Log-rank analysis was used to assess the significance of differences between curves for patients groups. Patients' characteristics were subjected to univariate analysis using log-rank test, before being entered into Cox proportional hazards (multivariate analyses) regression models, to determine prognostic factors (two sided). A *P* = 0.05 was considered significant. The studied prognostic factors are the gender, the sex, the stage, the B symptoms, the site, the LDH level, the bulky disease, the PS, and the epidural involvement.

All statistical analyses were conducted with the statistical package for social sciences (SPSS Inc., Chicago, IL).

## 3. Results

Twenty-six patients with PBL were included in GOELAMS 02 (19/26 patients, 73%) and 03 (7/26 patients, 37%) trials from 1986 to 1998 and their characteristics are summarized in Table 1.

Their median age was 46 years (range 17–69) (19 patients) and 70 years (range 65–75) (7 patients) in the GOELAMS 02 and 03 trials (cutoff of 60 years), respectively. Three patients between 63 and 69 years old but with excellent PS were included in the GOELAMS 02 trial by the investigators. Overall male/female ratio was 1.6.

The main PBL site was the axial skeleton. Two patients had bifocal bones lesions (axial and peripheral skeleton bone lesions). Skin and subcutaneous tissues were also involved in two patients. According to the Ann Arbor classification, 81% of patients were stage I and 19% were stage II. Six patients of the GOELAMS 02 trial had epidural involvement, revealed by paraplegia which may largely explain why PS was ≥2 for 35% of the patients. Predominant histological subtypes were diffuse, small cleaved cell lymphoma and diffuse, mixed small and large cell according to the Working Formulation, which correspond to DLBCL in the Working Health Organization classification.

Except for age and bulky disease, the two trials were comparable for histological type, site, Ann Arbor classification, B symptoms, and LDH (Table 1). All but one patient achieved CR (96%). A 63-year-old patient with VCAP-resistant costal disease died of progressive disease after 15 months. Eight (30%) relapses occurred at a median of 2.3 years (range 0.4–6.5) after CR: two relapses in the group of the 7 older patients and 6 in this of the 19 younger patients, which also included 65- and 69-year-old patients. Two out of the 8 relapses occurred at the initial PBL site.

Bulky disease was observed in nine patients and four of them relapsed. Four relapses occurred less than 2 years after CR, three between 2.1 and 5 years and one 6.5 years after CR. Among the six patients with epidural involvement, three relapsed (50%) and three were in sustained CR: two of these three relapses occurred *in situ*.

With median follow-up of 8 years (range 1.2–17), OS, EFS, and RFS were 64% ± 12, 62% ± 10, and 65% ± 10 years, respectively. OS, EFS, and RFS in the GOELAM 02 and 6 03 trials were, respectively, 66% ± 13, 59% ± 21, 62% ± 12 and 64% ± 21, 71% ± 14, 71% ± 17 with no significant difference between the two study groups (Table 2).

**Figure 1:**
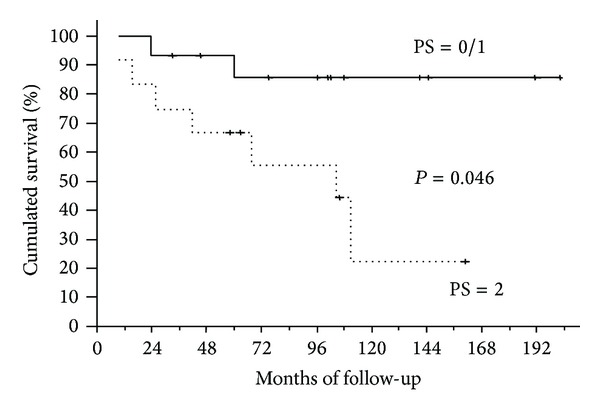
Overall survival curves of adults with non-Hodgkin primary bone lymphoma as a function of their performance status (PS).

According to univariate and multivariate analysis, poor PS (≥2) was associated with significantly shorter OS and EFS (Figure 1 and Table 2).

## 4. Discussion 

This report summarizes the results of a prospective study that evaluated the long-term outcome of 26 non-Hodgkin's PBL in adults after high-dose or conventional anthracycline-cyclophosphamide regimen combined with radiotherapy. Because of PBL rarity [1–4] and the heterogeneity of clinical procedure applied for diagnosis, staging, and treatment, controversies persist and no specific guidelines have been established.

The predominant histological profile of diffuse large B-cell lymphomas observed herein is consistent with published data [9, 10, 12]. No significant survival difference among between PLB subtypes has been observed in the literature [12, 13]. The median age was slightly higher than those reported previously (45–50 years) [3, 9, 14], which may reflect inclusion criteria. Unlike the majority of reports [1, 13, 15, 16], age did not influence the survival parameters (response rate, relapses, OS, RFS, progression, or EFS) of our patients. Indeed, the relatively good tolerance of treatment probably reflects the modulation of the chemotherapy dose according to age. Moreover, no radiotherapy complications were observed, certainly because of the low radiation dose delivered.

PBL was mostly diagnosed at stages I and II [9] except in one study with more stage IV with vertebral localization [17]. The stage appeared as the most important prognostic variable [1, 6, 8, 18]. The standard staging procedures (including bone radiographic, bone scan, and bone magnetic resonance imaging) may have underestimated the Ann Arbor staging. They do not allow an evaluation of the entire skeleton which is now optimately performed by using positron emission tomography [19]. Indeed stage IV is a factor of poor prognosis and probably requires an intensified treatment. An undervaluation of the stage leads to an insufficient treatment.

Peripheral skeleton is the most common site of PBL. However, one previous study reported high frequency of axial skeleton locations [20], which is a remarkable characteristic of our patients. Nevertheless PBL site did not influence OS and EFS in our study. However, some discrepancies concerning the definition of the axial involvement in the literature make it difficult to distinguish between the prognoses of axial skeleton versus limb involvement [2, 20].

Bulky disease was observed in 9 patients (all included in the VCAP group), 3 of whom having paraplegia. A pejorative impact of paraplegia has been suggested once [21]. Our univariate analysis identified a significantly unfavorable impact of PS ≥2 (OS, EFS). Our multivariate analysis retained poor PS but not epidural extension as being significantly associated with shorter EFS and OS. This observation is probably explained by relative redundancy between epidural extension and PS, since, quite frequently in neoplasic situations, paraplegic patients had PS ≥2.

Once, the standard treatment for localized disease primarily consisted of radiotherapy alone that is, from 40–60 Gy delivered within 4–6 weeks. Radiotherapy achieved high levels of local control (80–100%) but was followed by a high late relapse rate (50%) [5]. Then different CMT schedules of chemotherapy and radiotherapy were proposed [7, 15, 16, 22, 23]. It has been shown that anthracycline-based therapy improves the response rate and prolongs OS of patients with localized lymphomas [23]. In combination with radiotherapy is superior to radiotherapy regarding RFS and OS. this is may be explained by less relapse in the combined treatment group [23]. Thus, the general PBL reputation of poor prognosis no longer seems justified compared to other extranodal lymphomas [2, 4, 9, 10, 24]. Notably, the OS of disseminated PBL, that is, PBL with multiple locations treated with CMT, was higher than for localized-stage disease in some studies [15, 16, 25].

Our results confirmed the efficacy of CMT. The 96% CR rate is excellent, and mean 5- and 10-year OS reached 79% ± 8 (22 patients) and 63% ± 12 (19 patients), respectively. The 5- and 10-RFS at 70% ± 9 and 64% ± 10, respectively, were similar for both GOELAMS trials. These outcomes are not worse than those for 325 localized aggressive NHL that were treated in another GOELAMS trial [26] and confirm data suggested by comparable—although generally not randomized—published studies [6, 7, 13, 15, 16, 23]. We did not observe any significant difference between the stages I and II or normal versus high LDH levels. However, some prognostic criteria for localized aggressive NHL do not apply to PBL [2]. In our study, modulation of the anthracycline-cyclophosphamide dose did not significantly influence survival parameters (response, relapses, death, OS, RFS, progression, or EFS) for either group.

We observed a 30% relapse rate. Among them, seven relapses concerned pelvic localization, including three patients with paraplegia. A higher relapse rate of axial localization was reported previously [14, 27], leading once to a poor OS [14]. Although local control appears to be good, we think that systemic treatment can be further improved. Because of the period of recruitment, the potential benefit of adding an anti-CD20 monoclonal antibody, which seemed to be advantageous against PBL in one study [28], could not be tested here. Increasing the number of chemotherapy cycles alternating regimen cycles is another option.

The high paraplegia rate (6 patients, 23%) observed in our population merits attention. Epidural localization with paraplegia was associated with shorter OS (33% ± 25) but not statistically significant (OS of patients without paraplegia 75% ± 11, *P* = 0.18). However, of the seven deaths recorded, three were of patients with paraplegia. Among the six patients with paraplegia, three relapsed and two of these three relapses occurred *in situ*. Only 25% of the patients without epidural involvement relapsed. The poor prognosis of epidural PBL was reported in several studies [13, 29] with only one exception [30].

In summary, our results confirmed the efficacy of CMT against localized PBL. The systemic arm of CMT remains insufficient, in light of the high late relapse rate. The two schedules, the conventional VCEP-b and the high dose VCAP chemotherapy, are different in terms of drug dosages and for the type of anthracycline and the absence of bleomycin. VCEP-bleomycin regimen is effective, tolerated for older patients and high-dose anthracycline-cyclophosphamide did not improve the outcome.

But because of the period of recruitment of our prospective study, the potential benefit of an anti-CD20 monoclonal antibody is not tested. However the majority of PBL histology is B phenotype. Since 10 years, in these situations, the standard of chemotherapy included an anti-CD20 monoclonal antibody. The management of limited stage I-II PBL probably consists of 3 to 4 cycles of chemotherapy adding an anti-CD20 monoclonal antibody. More chemotherapy cycles should be considered for the patients with a high IPI score, even if the impact of IPI score is not yet validated in PBL. Radiotherapy is valid for local control and intensification remains discussed in the localized stages. But the staging must be precise. The staging procedure should now include positron emission tomography (PET) to see the entire skeleton, what it is not the case of our study. Furthermore, for bone lymphoma, the assessment of CR with CT scan is one major problem, the use of the PET allowed to solve.

On the other hand, epidural disease and PS ≥2 are factors of poor prognosis. New therapeutic strategies should be considered for these patients: addition of anti-CD20 monoclonal antibody, more chemotherapy cycles, and/or their alternation and intensification.

## 5. Clinical Practice Point

1. Treatment guidelines do not exist in PBL, because it is a rare entity and thus randomized prospective clinical trials are lacking. Nevertheless, combined chemotherapy and radiotherapy strategies have improved the management of PBL in particular in cases of advanced disease.
2. In this study, we review the clinical outcome of 26 patients with previously untreated PBL, all receiving anthracycline-cyclophosphamide containing regimen and consolidative radiation therapy. With median follow-up of 8 years, overall survival, event-free survival, and relapse-free survival were, respectively, 64%, 62%, and 65%. Poor PS was associated with shorter OS and EFS. High dose anthracycline-cyclophosphamide did not improve outcome.
3. In our opinion, combined chemotherapy and radiotherapy, nowadays probably in association with monoclonal anti-CD20 infusion, are efficace treatment for PBL. Intensified treatment must be considered for patients with PS ≥2. Moreover, new staging procedure including positron emission tomography should be now included.

## Figures and Tables

**Table 1:** Characteristics of 26 adults with non-Hodgkin primary bone lymphoma.

	No.	%
Trial		
02	19	73
03	7	27
Gender		
Male	16	61
Female	10	39
Stage		
I	21	81
II	5	19
B symptoms		
No	21	81
Yes	5	19
Site		
Axial skeleton	20	59
Spine	13	50
Pelvis	5	19
Rib	2	7
Peripheral skeleton	10	28
Limbs	6	23
Tibia	1	4
Humerus	1	4
Scapula	2	8
Radius/ulna	1	4
Finger	1	4
Skull	4	12
Mandible	3	12
Occipital	1	4
Histology		
Diffuse, mixed, small, large	4	15
Diffuse, large cleaved or not	18	69
Large cell immunoblastic	3	12
Anaplastic large-cell ANA Ki+	1	4
Immunophenotyping		
B cells	14	54
T cells	1	4
ANA Ki+	1	4
Nonassessable	10	38
LDH > N	7/21	33
PS		
0	6	23
1	11	42
2	6	23
3	3	12
IPI score		
0	5	19
1	11	42
2	3	11
3	2	7
Non-assessable	5	19

LDH: lactatedehydrogenase; N: normal; PS: performance status; IPI: international prognostic index.

Except for age and bulky disease, the two trials were comparable (no statistical significance). Bulky disease was observed in 9 patients, all included in the trial 02 (*P* = 0.02).

Six patients of the GOELAMS 02 trial had epidural involvement, revealed by paraplegia.

**Table 2:** Univariate and multivariate analyses of characteristics affecting survival of adults with non-Hodgkin primary bone lymphoma.

Characteristics	Univariate analysis		Multivariate analysis
	mean ± SD	*P*	*P*
*10-year OS, % *			
Trial			
02	66 ± 13	ns	ns
03	64 ± 21		
Epidural extension			
No	75 ± 11	ns	ns
Yes	33 ± 25		
PS ≥2			
No	86 ± 10	0.046	ns
Yes	28 ± 21		
*10-year EFS, % *
Trial			
02	59 ± 21	ns	ns
03	71 ± 14		
Epidural extension			
No	66 ± 11	ns	ns
Yes	50 ± 20		
PS ≥2			
No	79 ± 11	0.038	0.018
Yes	33 ± 17		

OS: overall survival; PS: performance status; EFS: event-free survival; ns: not significant.

Other studied factors (gender, stage, B symptoms, site, lactatedehydrogenase level, and bulky disease) do not have significantly statistical prognosis.
